# Axon Diameter and Intra-Axonal Volume Fraction of the Corticospinal Tract in Idiopathic Normal Pressure Hydrocephalus Measured by Q-Space Imaging

**DOI:** 10.1371/journal.pone.0103842

**Published:** 2014-08-05

**Authors:** Kouhei Kamiya, Masaaki Hori, Masakazu Miyajima, Madoka Nakajima, Yuriko Suzuki, Koji Kamagata, Michimasa Suzuki, Hajime Arai, Kuni Ohtomo, Shigeki Aoki

**Affiliations:** 1 Department of Radiology, Juntendo University Graduate School of Medicine, Tokyo, Japan; 2 Department of Neurosurgery, Juntendo University Graduate School of Medicine, Tokyo, Japan; 3 Philips Electronics Japan, Ltd., Tokyo, Japan; 4 Department of Radiology, Graduate School of Medicine, the University of Tokyo, Tokyo, Japan; University of Minnesota, United States of America

## Abstract

**Purpose:**

Previous studies suggest that compression and stretching of the corticospinal tract (CST) potentially cause treatable gait disturbance in patients with idiopathic normal pressure hydrocephalus (iNPH). Measurement of axon diameter with diffusion MRI has recently been used to investigate microstructural alterations in neurological diseases. In this study, we investigated alterations in the axon diameter and intra-axonal fraction of the CST in iNPH by q-space imaging (QSI) analysis.

**Methods:**

Nineteen patients with iNPH and 10 age-matched controls were recruited. QSI data were obtained with a 3-T system by using a single-shot echo planar imaging sequence with the diffusion gradient applied parallel to the antero-posterior axis. By using a two-component low-q fit model, the root mean square displacements of intra-axonal space ( =  axon diameter) and intra-axonal volume fraction of the CST were calculated at the levels of the internal capsule and body of the lateral ventricle, respectively.

**Results:**

Wilcoxon's rank-sum test revealed a significant increase in CST intra-axonal volume fraction at the paraventricular level in patients (*p*<0.001), whereas no significant difference was observed in the axon diameter. At the level of the internal capsule, neither axon diameter nor intra-axonal volume fraction differed significantly between the two groups.

**Conclusion:**

Our results suggest that in patients with iNPH, the CST does not undergo irreversible axonal damage but is rather compressed and/or stretched owing to pressure from the enlarged ventricle. These analyses of axon diameter and intra-axonal fraction yield insights into microstructural alterations of the CST in iNPH.

## Introduction

Idiopathic normal pressure hydrocephalus (iNPH) is a clinical entity of unknown cause and is characterized by the triad of gait disturbance, cognitive deterioration, and urinary incontinence [1]. It is also associated with ventricular enlargement, flattening of high-convexity sulci, and periventricular T2-weighted image hyperintensity in the absence of elevated cerebrospinal fluid (CSF) pressure [2]. Gait disturbance is the most frequent symptom of iNPH [3] and is treated by CSF shunting [4]. Although the etiology of gait disturbance in iNPH is not completely understood, a plausible explanation is that the corticospinal tract (CST) is distorted by expansion of the lateral ventricles [1], [5], [6].

Diffusion tensor imaging (DTI) has been applied to neurological and psychological diseases and is useful to detect brain abnormalities that can not be recognized by conventional T1- or T2- weighted images [7], [8]. Previous studies conducted with DTI revealed increases of fractional anisotropy (FA) and axial diffusivity values in the CST in patients with iNPH [9]–[17], which tended to return to normal after placement of a ventriculoperitoneal (VP) shunt [9]–[12]. The increases in FA and axial diffusivity have been suggested to result from ventricular enlargement that mechanically compresses the tract and yields more directional water diffusion along it. Diffusion MRI is expected to become a non-invasive method for diagnosing iNPH and predicting the response to surgery [12], [17].

Q-space imaging (QSI), a diffusion MRI technique that does not assume that the displacement probability of diffusing water molecules has a Gaussian distribution, can provide quantitative tissue architecture information at cellular dimensions [18]–[22]. Recently, analysis of axon diameter of neural fibers by using diffusion MRI is becoming a topic for investigation of microstructural alteration in neurological disease [23]–[28], although assessment of axonal architecture usually requires high gradient amplitudes and long scanning times, which are not clinically applicable. A two-component low-q fit model for QSI analysis, proposed by Ong et al. [29], enables measurement of the axon diameters of neural fibers with a reasonable scanning time. Briefly, QSI provides a molecular displacement probability density function (PDF), which reflects the axonal architecture, such as axon membranes and myelin sheath acting as barriers to diffusing molecules. In the white matter, the dominant diffusion barrier is the axonal membrane, and the spacing between barriers can be regarded as mean axonal diameter [19]. A two-component low-q fit model used in this study has the following two merits; 1) it accounts for signal from extra- and intra-axonal spaces and has better correlations with pathological findings than a single-component model, 2) it does not require very high gradient amplitudes [29]. The limitation of this method is that it requires prior knowledge of the fiber orientation, because the diffusion gradient must be applied perpendicular to the fiber direction.

The purpose of this study is to investigate alterations in the axonal architecture of the CST in patients with iNPH by using a two-component low-q fit analysis of QSI.

## Materials and Methods

### Ethics Statement

This study was conducted in accordance with the Declaration of Helsinki and approved by the Institutional Review Board of Juntendo University Hospital and all persons gave their written informed consent prior to their inclusion in the study.

### Patients

Nineteen patients with iNPH (10 males and 9 females; 74.3±6.2 years old) and 10 age-matched control subjects (3 males and 7 females; 75.8±5.2 years old) were recruited. Diagnosis of iNPH was made according to the diagnostic criteria of probable iNPH [30]. Those who had a history of neurological disease or any significant findings (as observed on routine MR images) that might affect the brain were excluded. Normal control subjects were required to be >60 years of age and have no neurological or psychological symptoms, history of neurologic diseases, or apparent abnormalities observed on conventional MR images.

### QSI data acquisition and processing

QSI data were obtained with a 3-T unit (Achieva, Philips Healthcare, Best, The Netherlands) by using a single-shot echo planar imaging (EPI) sequence. The patient was positioned so that the anterior commissure–posterior commissure (AC-PC) line is parallel to the scanner's x-y plane. The scan parameters were: repetition rate/echo time (TR/TE)  = 4500/99 ms, field of view (FOV)  = 240×240 mm^2^, matrix size  = 96×96, slice thickness  = 5 mm, 10 axial sections including the level of internal capsule, number of excitations (NEX)  = 2, half-Fourier factor  = 0.667, 16 b-values (0, 1000, 2000, … 15000 s/mm^2^, applied in sequential order), and acquisition time  = 828 s. The gradient duration (δ) and time between the two leading edges of the diffusion gradient (Δ) were 39.3 and 48.7 ms, respectively. We did not apply any distortion corrections, because correction was difficult especially at high b values and resulted in severe signal defect in some of the patients.

**Figure 1:**
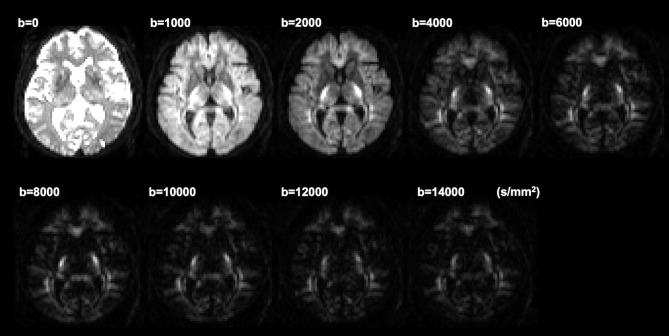
Examples of the acquired diffusion weighted images.

The two-component low-q fit method for axon diameter analysis necessitates a diffusion gradient perpendicular to the fiber tract to be measured. Ideally, the appropriate direction of diffusion gradient can be determined in each patient by performing tractography of the CST, though it is not realistic in clinical examinations. Instead, we applied diffusion gradient parallel to the antero-posterior axis of the scanner's coordinate system, as the known course of CST [52], [53] is substantially perpendicular to the scanner's x-y plane. Examples of the acquired images are shown in Figure 1. By applying the diffusion gradient parallel to the antero-posterior axis, the CST could be identified as a hyperintense tract running from the precentral gyrus to the cerebral peduncle through the posterior limb of the internal capsule [31]. Each ROI was placed manually so that it includes the brightly-appearing CST, using b = 1000∼4000 s/mm^2^ images. The cranial and caudal sections were also used as references for continuity of the tract. Measurements were performed at two levels. The first section was selected so that it contains the posterior limb of the internal capsule. The second one was selected as two or three sections cranial to the first one, where the CST runs closest to the lateral ventricle. By using in-house software developed in Matlab (R2011b; MathWorks, Natick, MA, USA), the root mean square displacements (RMSDs) of the intra-axonal space ( =  axon diameter) and intra-axonal volume fraction of the CST were calculated by fitting the echo attenuations (normalized to the maximum value at the q = 0) to equation (1) with a nonlinear least squares algorithm: E(q) = (1−f_I_) exp(−2π^2^q^2^Z_E_^2^)+f_I_ exp(−2π^2^q^2^Z_I_^2^) …(1) where f_I_ is the relaxation-weighted intra-axonal volume fraction, and Z_E_ and Z_I_ are the RMSDs of diffusing molecules in the extra- and intra-axonal spaces, respectively.

### Statistical analysis

Statistical analyses were performed by using JMP software (ver. 10.0.2; SAS Institute Inc. Cary, NC, USA). The axon diameter and intra-axonal volume fraction values of the CST from both hemispheres were compared between the patients and controls. To minimize type I errors with multiple comparisons, Bonferroni's correction was applied. The significance level (p = 0.05) was therefore reduced to an adjusted p level of 0.006.

## Results

**Figure 2:**
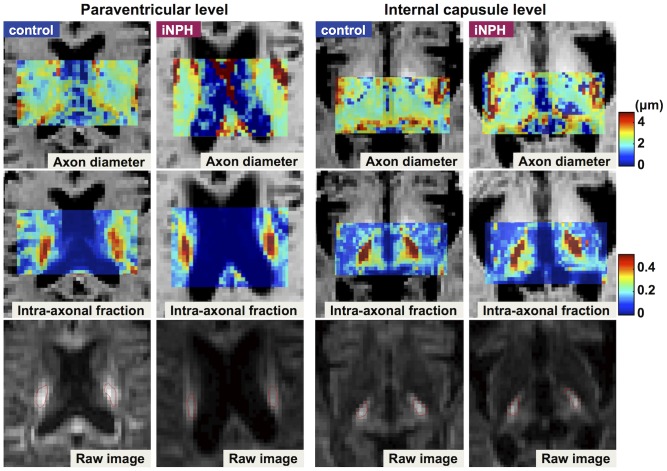
Examples of axon diameter maps (*top row*) and intra-axonal volume fraction maps (*middle row*). ROIs are shown in the raw diffusion image (*bottom row*, b = 2000 s/mm^2^).

**Figure 3:**
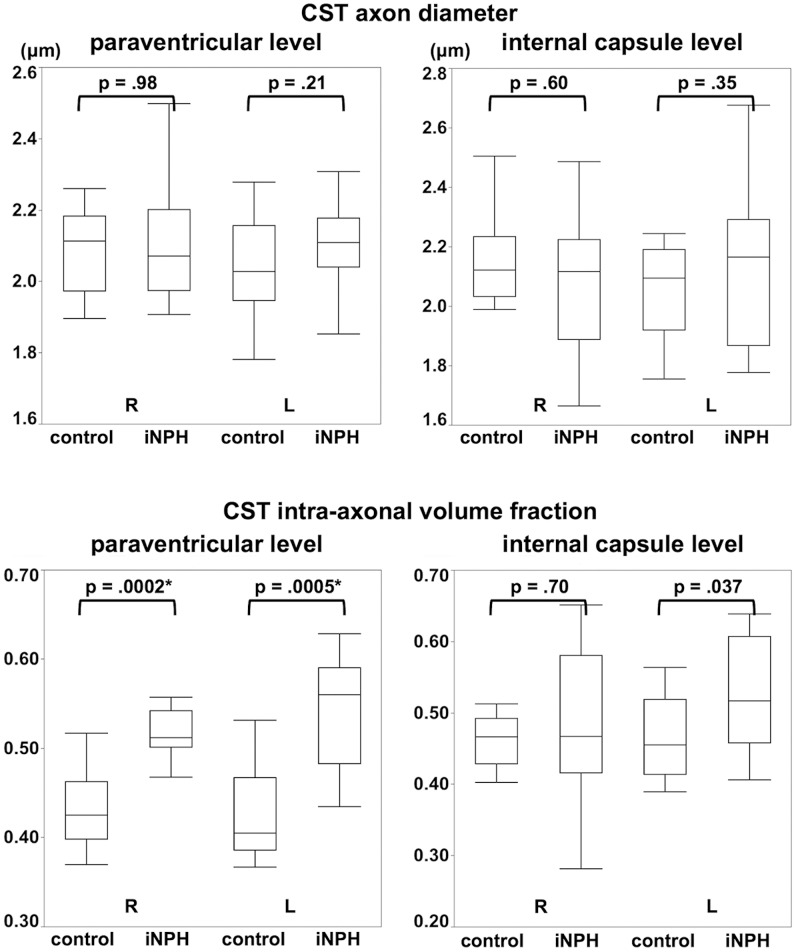
Boxplot comparing the CST axon diameter and intra-axonal volume fraction between the controls and iNPH patients. Statistical analyses revealed a significant increase in CST intra-axonal volume fraction at the paraventricular level in the patients, whereas no significant difference was observed in the axon diameter. At the level of the internal capsule, neither axon diameter nor intra-axonal volume fraction differed significantly between the two groups. * The significance level was set at *p* = 0.006 (Bonferroni's correction for multiple comparison).

Excellent fitting was obtained in all ROIs (R^2^>0.95). Shapiro-Wilk's test was performed to test the hypothesis that the data satisfied Gaussian distribution (the significance level was set at *p* = 0.05). As it was revealed that the assumption of Gaussian distribution was not satisfied in the measurements of axon diameter at the internal capsule level (p = 0.004), Wilcoxon's rank-sum test was used for the following group analyses. At the paraventricular level, the CST intra-axonal volume fraction was significantly higher in patients with iNPH than in the controls (right, 0.43±0.04 for the controls, 0.53±0.05 for the patients, *p* = 0.0002; left, 0.43±0.06 for the controls, 0.54±0.06 for the patients, *p* = 0.0005), whereas no significant difference was observed in the CST axon diameter. At the level of the internal capsule, no significant differences were observed between the two groups in either axon diameter or intra-axonal volume fraction (Table 1, Figs. 2, 3). There were no statistically significant differences in ROI sizes between the patients and the controls (Student's t-test; the internal capsule level, 43.6±4.8 mm^2^ for the controls and 43.3±7.3 mm^2^ for the patients, *p* = 0.83; the paraventricular level, 43.0±9.6 mm^2^ for the controls and 41.9±7.4 mm^2^ for the patients, *p* = 0.66)

**Table 1:** Results of the measurement of CST axon diameter and intra-axonal volume fraction.

	controls	iNPH patients
	right	left	right	left
Internal capsule level				
axon diameter (µm)	2.07±0.35	2.00±0.31	2.00±0.53	2.16±0.28
intra-axonal volume fraction	0.47±0.05	0.46±0.06	0.49±0.09	0.53±0.08
Paraventricular level				
axon diameter (µm)	2.09±0.12	2.04±0.14	2.10±0.16	2.11±0.15
intra-axonal volume fraction	0.43±0.04	0.43±0.06	0.53±0.05	0.54±0.06

## Discussion

A two-component low-q fit analysis of QSI revealed that the CST intra-axonal volume fraction in areas near the ventricles was increased in iNPH patients compared with controls, whereas the CST axon diameter was unaltered. Neither CST axon diameter nor intra-axonal volume fraction differed significantly at the level of the internal capsule. Our results are in line with previous DTI studies showing increased diffusion anisotropy of the CST in iNPH, presumably due to compaction of neuronal fibers [9]–[17]. The increase in CST intra-axonal volume fraction was limited to areas near the lateral ventricle in our study, suggesting that it results from compression by the enlarged ventricles. The unaltered axon diameter of the CST suggests that the iNPH patients involved in this study did not have irreversible axonal damage of the CST.

The exact pathogenesis of the gait disturbance in iNPH is not entirely understood. Though our results suggest that the axons are densely packed in the CST with reduced extra-axonal space, there is no readily available explanation why such situation results in the characteristic gait disturbance in iNPH. A classical hypothesis that remains plausible is that the CST is compressed and/or deformed because of enlargement of the lateral ventricles [1], [5], [6]. Other pathological changes observed in the brains of patients with iNPH, such as ischemia and gliosis due to transependymal diapedesis of the CSF, may to some extent be related to gait disturbance [32]–[36]. However, the reversibility of symptoms after shunt surgery even after a long period suggests that irreversible axonal damage is unlikely to be the sole cause of gait disturbance [4]. The “compression hypothesis” is also supported by the fact that the fibers of the legs are closest in proximity to the lateral ventricles [37]–[39]; this explains why gait disturbance is the most prominent neurological feature in iNPH. Moreover, a detailed tract-specific analysis of the CST demonstrated that the increase in FA was limited to areas near the lateral ventricle [15], an observation consistent with CST compression by the ventricular enlargement.

Analyses of the axon diameter and axon density by using diffusion MRI could have a significant impact on our understanding of white matter architecture and connectivity, neuroanatomical changes that occur in white matter disorders, and changes that occur in white matter during normal and abnormal development. These indices are more straightforward and easier to interpret than other diffusion metrics, such as the mean diffusivity, fractional anisotropy, directional diffusivity, and directional kurtosis, each of which must be interpreted in combination with one or more of the others to understand the microstructural changes [40], [41]. Because axon diameter determines conduction velocity, this metric and the axon density provide information about the role and performance of white matter pathways [42]–[44]. Axon diameter analysis would also provide a means of testing hypotheses that assume changes in the diameter distribution in diseases such as amyotrophic lateral sclerosis [45], multiple sclerosis [46], and autism [47].

The present results need to be interpreted carefully and hopefully validated by more dedicated experiments, because the short gradient pulse (SGP) approximation (Δ>>δ) was not satisfied in this study. In principal, SGP approximation needs to be fulfilled for accurate compartment size measurement by QSI. However, with clinical scanners, high q-values can only be obtained with long diffusion gradient pulses because of the relatively weak gradients [20], [48], [49]. The previous experimental studies reported that the diffraction minima is pushed towards higher q values and the extracted compartment size becomes smaller than the real size when the SGP approximation is violated (Δ/δ∼1) [50]. Though the situation becomes more complicated when there are more than two compartments [50], we speculate that the intra-axonal volume fraction in this study is larger than the real value, as the echo attenuation curve vs q values shifts to the right. Mathematical calibration with the ideal Δ/δ settings [50], or the use of a double-pulsed gradient sequence [51], may be useful to overcome this issue.

The other limitations to this study include the following. First, interpretation of the increase in intra-axonal volume fraction is somewhat ambiguous as it is not directly equal to axonal density. Though we speculate the increased intra-axonal volume fraction reflects that the neural fibers are densely packed with reduced extra-axonal space, other conditions, such as changes in the distribution of axon diameter (i.e, increase of large- and small-diameter axons with few middle-sized axons), may yield similar results. In addition, the intra-axonal volume fraction obtained by the two-component low-q fit method is not completely proven to correlate with that obtained from pathological analyses [29]. Second, the determination of diffusion gradient direction was approximative, and it could have been slightly different from what it should be (perpendicular to the CST). Also, previous DTI studies in iNPH have typically reported increased FA in the CST and decreased FA in the corpus callosum, suggesting regionally dependent microstructural alterations [9], [11], [13], [15]. Therefore, our results require validation by an orientationally invariant method for measuring the axon diameter [25], [54]. Lastly, owing to the small sample size and lack of post-operative imaging, the clinical relevance of the QSI measures, such as in monitoring the effect of surgery or pre-operatively predicting the response to surgery, could not be established.

## Conclusions

In this study, an analysis of axon diameter and intra-axonal volume fraction demonstrated that in patients with iNPH, the CST is compressed by the ventricular enlargement but does not undergo irreversible axonal damage. The axon diameter and intra-axonal volume fraction obtained by QSI yield insights into microstructural alterations in iNPH. Their potential use in predicting the response to surgery or in post-operative monitoring requires further investigation.

## References

[1] Relkin N, Marmarou A, Klinge P, Bergsneider M, Black PM (2005) Diagnosing idiopathic normal-pressure hydrocephalus. Neurosurgery 57: : S4–16; discussion ii–v. 10.1227/01.neu.0000168185.29659.c5 16160425

[2] Sasaki M, Honda S, Yuasa T, Iwamura A, Shibata E, et al (2008) Narrow CSF space at high convexity and high midline areas in idiopathic normal pressure hydrocephalus detected by axial and coronal MRI. Neuroradiology 50: 117–122. 17992524 10.1007/s00234-007-0318-x

[3] Marmarou A, Young HF, Aygok GA, Sawauchi S, Tsuji O, et al (2005) Diagnosis and management of idiopathic normal-pressure hydrocephalus: a prospective study in 151 patients. Journal of Neurosurgery 102: 987–997. 16028756 10.3171/jns.2005.102.6.0987

[4] Meier U, Lemcke J (2006) Clinical outcome of patients with idiopathic normal pressure hydrocephalus three years after shunt implantation. Acta Neurochir Suppl 96: 377–380. 10.1007/3-211-30714-1_78 16671489

[5] Hakim S, Venegas JG, Burton JD (1976) The physics of the cranial cavity, hydrocephalus and normal pressure hydrocephalus: mechanical interpretation and mathematical model. Surgical Neurology 5: 187–210. 1257894

[6] Adams RD, Fisher CM, Hakim S, Ojemann RG, Sweet WH (1965) Symptomatic Occult Hydrocephalus with “Normal” Cerebrospinal-Fluid Pressure.A Treatable Syndrome. New England Journal of Medicine 273: 117–126. 14303656 10.1056/NEJM196507152730301

[7] Shizukuishi T, Abe O, Aoki S (2013) Diffusion tensor imaging analysis for psychiatric disorders. Magnetic resonance in medical science 12: 153–159. 10.2463/mrms.2012-0082 23857149

[8] Inglese M, Bester M (2010) Diffusion imaging in multiple sclerosis: research and clinical implications. NMR in Biomedicine 23: 865–872. 20882528 10.1002/nbm.1515 PMC3071990

[9] Assaf Y, Ben-Sira L, Constantini S, Chang LC, Beni-Adani L (2006) Diffusion tensor imaging in hydrocephalus: initial experience. AJNR American Journal of Neuroradiology 27: 1717–1724. 16971621 PMC8139798

[10] Jang SH, Ho Kim S (2011) Diffusion tensor imaging following shunt in a patient with hydrocephalus. Journal of Neuroimaging 21: 69–72. 19555407 10.1111/j.1552-6569.2009.00394.x

[11] Scheel M, Diekhoff T, Sprung C, Hoffmann KT (2012) Diffusion tensor imaging in hydrocephalus–findings before and after shunt surgery. Acta Neurochirurgica 154: 1699–1706. 22610531 10.1007/s00701-012-1377-2

[12] Jurcoane A, Keil F, Szelenyi A, Pfeilschifter W, Singer OC, et al.. (2013) Directional diffusion of corticospinal tract supports therapy decisions in idiopathic normal-pressure hydrocephalus. Neuroradiology. 10.1007/s00234-013-1289-8 24158631

[13] Hattingen E, Jurcoane A, Melber J, Blasel S, Zanella FE, et al (2010) Diffusion tensor imaging in patients with adult chronic idiopathic hydrocephalus. Neurosurgery 66: 917–924. 20404696 10.1227/01.NEU.0000367801.35654.EC

[14] Hattori T, Yuasa T, Aoki S, Sato R, Sawaura H, et al (2011) Altered microstructure in corticospinal tract in idiopathic normal pressure hydrocephalus: comparison with Alzheimer disease and Parkinson disease with dementia. AJNR American Journal of Neuroradiology 32: 1681–1687. 21816921 10.3174/ajnr.A2570 PMC7965405

[15] Hattori T, Ito K, Aoki S, Yuasa T, Sato R, et al (2012) White matter alteration in idiopathic normal pressure hydrocephalus: tract-based spatial statistics study. AJNR American Journal of Neuroradiology 33: 97–103. 22016412 10.3174/ajnr.A2706 PMC7966161

[16] Nakanishi A, Fukunaga I, Hori M, Masutani Y, Takaaki H, et al (2013) Microstructural changes of the corticospinal tract in idiopathic normal pressure hydrocephalus: a comparison of diffusion tensor and diffusional kurtosis imaging. Neuroradiology 55: 971–976. 23728069 10.1007/s00234-013-1201-6

[17] Kim MJ, Seo SW, Lee KM, Kim ST, Lee JI, et al (2011) Differential diagnosis of idiopathic normal pressure hydrocephalus from other dementias using diffusion tensor imaging. AJNR American Journal of Neuroradiology 32: 1496–1503. 21700790 10.3174/ajnr.A2531 PMC7964370

[18] Cohen Y, Assaf Y (2002) High b-value q-space analyzed diffusion-weighted MRS and MRI in neuronal tissues - a technical review. NMR in Biomedicine 15: 516–542. 12489099 10.1002/nbm.778

[19] Assaf Y, Mayk A, Cohen Y (2000) Displacement imaging of spinal cord using q-space diffusion-weighted MRI. Magnetic Resonance in Medicine 44: 713–722. 11064406 10.1002/1522-2594(200011)44:5<713::aid-mrm9>3.0.co;2-6

[20] Farrell JA, Smith SA, Gordon-Lipkin EM, Reich DS, Calabresi PA, et al (2008) High b-value q-space diffusion-weighted MRI of the human cervical spinal cord in vivo: feasibility and application to multiple sclerosis. Magnetic Resonance in Medicine 59: 1079–1089. 18429023 10.1002/mrm.21563 PMC2849312

[21] Farrell JA, Zhang J, Jones MV, Deboy CA, Hoffman PN, et al (2010) q-space and conventional diffusion imaging of axon and myelin damage in the rat spinal cord after axotomy. Magnetic Resonance in Medicine 63: 1323–1335. 20432303 10.1002/mrm.22389 PMC2862595

[22] Hori M, Fukunaga I, Masutani Y, Taoka T, Kamagata K, et al (2012) Visualizing non-Gaussian diffusion: clinical application of q-space imaging and diffusional kurtosis imaging of the brain and spine. Magn Reson Med Sci 11: 221–233. 23269009 10.2463/mrms.11.221

[23] Assaf Y, Blumenfeld-Katzir T, Yovel Y, Basser PJ (2008) AxCaliber: a method for measuring axon diameter distribution from diffusion MRI. Magnetic Resonance in Medicine 59: 1347–1354. 18506799 10.1002/mrm.21577 PMC4667732

[24] Dyrby TB, Sogaard LV, Hall MG, Ptito M, Alexander DC (2013) Contrast and stability of the axon diameter index from microstructure imaging with diffusion MRI. Magnetic Resonance in Medicine 70: 711–721. 23023798 10.1002/mrm.24501 PMC4199276

[25] Alexander DC, Hubbard PL, Hall MG, Moore EA, Ptito M, et al (2010) Orientationally invariant indices of axon diameter and density from diffusion MRI. Neuroimage 52: 1374–1389. 20580932 10.1016/j.neuroimage.2010.05.043

[26] Assaf Y, Alexander DC, Jones DK, Bizzi A, Behrens TE, et al (2013) The CONNECT project: Combining macro- and micro-structure. Neuroimage 80: 273–282. 23727318 10.1016/j.neuroimage.2013.05.055

[27] McNab JA, Edlow BL, Witzel T, Huang SY, Bhat H, et al (2013) The Human Connectome Project and beyond: initial applications of 300 mT/m gradients. Neuroimage 80: 234–245. 23711537 10.1016/j.neuroimage.2013.05.074 PMC3812060

[28] Morozov D, Bar L, Sochen N, Cohen Y (2013) Modeling of the diffusion MR signal in calibrated model systems and nerves. NMR in Biomedicine 26: 1787–1795. 24105913 10.1002/nbm.3018

[29] Ong HH, Wehrli FW (2010) Quantifying axon diameter and intra-cellular volume fraction in excised mouse spinal cord with q-space imaging. Neuroimage 51: 1360–1366. 20350604 10.1016/j.neuroimage.2010.03.063 PMC2895496

[30] Mori E, Ishikawa M, Kato T, Kazui H, Miyake H, et al (2012) Guidelines for management of idiopathic normal pressure hydrocephalus: second edition. Neurologia Medico-Chirurgica 52: 775–809. 23183074 10.2176/nmc.52.775

[31] Schaefer PW, Grant PE, Gonzalez RG (2000) Diffusion-weighted MR imaging of the brain. Radiology 217: 331–345. 11058626 10.1148/radiology.217.2.r00nv24331

[32] Akai K, Uchigasaki S, Tanaka U, Komatsu A (1987) Normal pressure hydrocephalus. Neuropathological study. Acta Pathologica Japonica 37: 97–110. 3577765

[33] Del Bigio MR (1993) Neuropathological changes caused by hydrocephalus. Acta Neuropathol 85: 573–585. 8337936 10.1007/BF00334666

[34] Di Rocco C, Di Trapani G, Maira G, Bentivoglio M, Macchi G, et al (1977) Anatomo-clinical correlations in normotensive hydrocephalus. Reports on three cases. Journal of the Neurological Sciences 33: 437–452. 915528 10.1016/0022-510x(77)90139-3

[35] Bradley WG Jr, Whittemore AR, Watanabe AS, Davis SJ, Teresi LM, et al (1991) Association of deep white matter infarction with chronic communicating hydrocephalus: implications regarding the possible origin of normal-pressure hydrocephalus. AJNR American Journal of Neuroradiology 12: 31–39. 1899515 PMC8367539

[36] Krauss JK, Regel JP, Vach W, Droste DW, Borremans JJ, et al (1996) Vascular risk factors and arteriosclerotic disease in idiopathic normal-pressure hydrocephalus of the elderly. Stroke 27: 24–29. 8553398 10.1161/01.str.27.1.24

[37] Kim JS, Pope A (2005) Somatotopically located motor fibers in corona radiata: evidence from subcortical small infarcts. Neurology 64: 1438–1440. 15851738 10.1212/01.WNL.0000158656.09335.E7

[38] Holodny AI, Gor DM, Watts R, Gutin PH, Ulug AM (2005) Diffusion-tensor MR tractography of somatotopic organization of corticospinal tracts in the internal capsule: initial anatomic results in contradistinction to prior reports. Radiology 234: 649–653. 15665224 10.1148/radiol.2343032087

[39] Zolal A, Vachata P, Hejcl A, Bartos R, Malucelli A, et al (2012) Anatomy of the supraventricular portion of the pyramidal tract. Acta Neurochirurgica 154: 1097–1104 discussion 1104. 22527572 10.1007/s00701-012-1326-0

[40] Jensen JH, Helpern JA (2010) MRI quantification of non-Gaussian water diffusion by kurtosis analysis. NMR in Biomedicine 23: 698–710. 20632416 10.1002/nbm.1518 PMC2997680

[41] Wu EX, Cheung MM (2010) MR diffusion kurtosis imaging for neural tissue characterization. NMR in Biomedicine 23: 836–848. 20623793 10.1002/nbm.1506

[42] Ritchie JM (1982) On the relation between fibre diameter and conduction velocity in myelinated nerve fibres. Proceedings of the Royal Society of London Series B: Biological Sciences 217: 29–35. 10.1098/rspb.1982.0092 6131421

[43] Aboitiz F, Scheibel AB, Fisher RS, Zaidel E (1992) Fiber composition of the human corpus callosum. Brain Research 598: 143–153. 1486477 10.1016/0006-8993(92)90178-c

[44] Lamantia AS, Rakic P (1990) Cytological and quantitative characteristics of four cerebral commissures in the rhesus monkey. Journal of Comparative Neurology 291: 520–537. 2329189 10.1002/cne.902910404

[45] Cluskey S, Ramsden DB (2001) Mechanisms of neurodegeneration in amyotrophic lateral sclerosis. Molecular Pathology 54: 386–392. 11724913 PMC1187128

[46] DeLuca GC, Ebers GC, Esiri MM (2004) Axonal loss in multiple sclerosis: a pathological survey of the corticospinal and sensory tracts. Brain 127: 1009–1018. 15047586 10.1093/brain/awh118

[47] Conturo TE, Williams DL, Smith CD, Gultepe E, Akbudak E, et al (2008) Neuronal fiber pathway abnormalities in autism: an initial MRI diffusion tensor tracking study of hippocampo-fusiform and amygdalo-fusiform pathways. Journal of the International Neuropsychological Society 14: 933–946. 18954474 10.1017/S1355617708081381 PMC3298449

[48] Assaf Y, Ben-Bashat D, Chapman J, Peled S, Biton IE, et al (2002) High b-value q-space analyzed diffusion-weighted MRI: application to multiple sclerosis. Magnetic Resonance in Medicine 47: 115–126. 11754450 10.1002/mrm.10040

[49] Mayzel-Oreg O, Assaf Y, Gigi A, Ben-Bashat D, Verchovsky R, et al (2007) High b-value diffusion imaging of dementia: application to vascular dementia and alzheimer disease. Journal of the Neurological Sciences 257: 105–113. 17360001 10.1016/j.jns.2007.01.048

[50] Bar-Shir A, Avram L, Ozarslan E, Basser PJ, Cohen Y (2008) The effect of the diffusion time and pulse gradient duration ratio on the diffraction pattern and the structural information estimated from q-space diffusion MR: experiments and simulations. Journal of Magnetic Resonance 194: 230–236. 18667345 10.1016/j.jmr.2008.07.009 PMC7477617

[51] Shemesh N, Ozarslan E, Basser PJ, Cohen Y (2009) Measuring small compartmental dimensions with low-q angular double-PGSE NMR: The effect of experimental parameters on signal decay. Journal of Magnetic Resonance 198: 15–23. 19186086 10.1016/j.jmr.2009.01.004

[52] Kunimatsu A, Aoki S, Masutani Y, Abe O, Mori H, et al (2003) Three-dimensional white matter tractography by diffusion tensor imaging in ischaemic stroke involving the corticospinal tract. Neuroradiology 45: 532–535. 12856090 10.1007/s00234-003-0974-4

[53] Yamada K, Kizu O, Kubota T, Ito H, Matsushima S, et al (2007) The pyramidal tract has a predictable course through the centrum semiovale: a diffusion-tensor based tractography study. Journal of Magnetic Resonance Imaging 26: 519–524. 17729353 10.1002/jmri.21006

[54] Barazany D, Jones D, Assaf Y (2011) AxCaliber 3D. Proc Int Soc Magn Reson Med 19: 76.

